# Susceptibility and plant immune control—a case of mycorrhizal strategy for plant colonization, symbiosis, and plant immune suppression

**DOI:** 10.3389/fmicb.2023.1178258

**Published:** 2023-07-05

**Authors:** Matthew Chekwube Enebe, Mariana Erasmus

**Affiliations:** Centre for Mineral Biogeochemistry, University of the Free State, Bloemfontein, South Africa

**Keywords:** mycorrhizal fungi (MF), disease susceptibility, ecological function, plants, immune control

## Abstract

Plants and microbes (mycorrhizal fungi to be precise) have evolved together over the past millions of years into an association that is mutualist. The plants supply the fungi with photosynthates and shelter, while the fungi reciprocate by enhancing nutrient and water uptake by the plants as well as, in some cases, control of soil-borne pathogens, but this fungi–plant association is not always beneficial. We argue that mycorrhizal fungi, despite contributing to plant nutrition, equally increase plant susceptibility to pathogens and herbivorous pests' infestation. Understanding of mycorrhizal fungi strategies for suppressing plant immunity, the phytohormones involved and the signaling pathways that aid them will enable the harnessing of tripartite (consisting of three biological systems)—plant–mycorrhizal fungi–microbe interactions for promoting sustainable production of crops.

## Introduction

Plants are sessile organisms whose growth and development depend on its interaction with the soil, environment, and microbes. The fixed nature of plants to the soil during its growth cycle has made its interactions with soil dwelling microbes a prerequisite phenomenon for survival. Inhabiting the soil are various groups of microbes that can exhibit either a parasitic lifestyle, commensal, saprophytic, or symbiotic relationships with the plant host. These microbes benefit from plants by obtaining organic carbon and in return could either positively enhance plant nutritional status, fitness, and wellbeing or could cause diseases to the plants. Either way, an effect is exerted on plants. To counteract these effects, plants over the years have evolved various defense strategies ranging from signaling, immune receptors, and enzymes to morphological structures that enable them to withstand pathogen attacks and positively interact with symbionts. In this evolutionary arm race, both pathogens and symbionts are devising counter measures against plant immune defenses to increase their fitness in plants (Kaur et al., 2022). For instance, pathogens are notorious for manipulating plant immune network by introducing effectors with the aid of type 3 secretion system and/or hormonal synthesis that dampen plant immunity and increase its vulnerability (Ronald and Joe, 2018; Shen et al., 2018; Lee et al., 2019). Through this approach, pathogens can establish themselves in their hosts and cause diseases.

Suffice to say, all microbes that interact with plants possess effectors that aid their aversion of plant immune attack and recognition since microbes (both symbionts and pathogens) possess unique cell wall-associated molecular structures known as microbe-associated molecular patterns that trigger immune responses in plants upon contact with plant pattern recognition receptors (Jones and Dangl, 2006), and mycorrhizal fungi are no exception. Mycorrhizal fungi, obligate biotrophic microbes, are symbionts, which establish a mutualistic relationship with the roots of terrestrial plants and perform various roles ranging from enhanced nutrient acquisition by plants, plant growth promotion, and tolerance to abiotic and biotic stresses (Yousef et al., 2016; Poveda et al., 2020; Rashad et al., 2021; Almario et al., 2022). Approximately 80% or more of terrestrial plants are colonized by mycorrhizal fungi (particularly arbuscular mycorrhizal fungi) (Smith and Read, 2008). Although not all parasitic fungi are pathogenic, mycorrhizal fungi and their biotrophic interaction with plants could be regarded as semi parasitic but not pathogenic fungi of plants since they obtain their nutrients from the living tissues of plants without interfering with the biological functions of their host. Like most pathogenic fungi, mycorrhizae equally trigger plant immune responses during infection and colonization. To achieve efficient colonization, mycorrhizal fungi induce the upregulation of endocytic effectors in host plants, which aid its accommodation by the plants. It equally stimulates the division of root cortical cells, a mechanism that enhances plant–fungi relationships (Russo et al., 2019). In addition, through the production of effectors such as SP7 that interact with ERF19 of the host and reduce the induction of ERF19-mediated defense gene expression, mycorrhizal fungi are able to colonize its host and prevent its destruction by plant immune arsenals (Kloppholz et al., 2011).

Crucially, despite the beneficial roles of mycorrhizal fungi in plant nutrition, growth promotion, and protection, infection and colonization of plants by these symbionts often result in a counterproductive outcome. Evidence has shown that through the suppression of plant immunity to enhance its colonization, mycorrhizal fungi indirectly exacerbate disease incidence in plants (Ross, 1972; Davis et al., 1978; Shaul et al., 1999; Wang et al., 2021). Several studies have focused light on the efficacy of arbuscular mycorrhizal fungi contributions to the biocontrol of plant pathogens such as nematodes, viruses, and bacteria through induction of plant immunity and activation of ethylene production (Duc and Posta, 2018; Miozzi et al., 2019; Poveda et al., 2020). For instance, Fujita et al. (2022) posited that through the induction of salicylic and jasmonic acid in mycorrhizal fungi-colonized tomato plants, both pathogenic fungi (*Botrytis cinerea*) and bacteria (*Pseudomonas syringae* pv. tomato DC3000) were prevented from establishing infections and diseases in the tomato plants. In another study, the incidence and disease severity caused by tomato mosaic virus in infected tomato plants were significantly reduced in mycorrhizal fungi-colonized plants, perhaps showing a protective role of arbuscular mycorrhizal fungi (Aseel et al., 2019). Readers interested in understanding the various mechanisms mycorrhizal fungi employ in enhancing plant tolerance to phytopathogens should refer to a current review by Dowarah et al. (2021). However, nonetheless, the consequences of these fungi–plant interactions on plant susceptibility to pests and pathogens are an area that seems to be receiving less attention.

In this review, we highlight the recent understanding of the consequences of mycorrhizal fungi interactions with plants on the development of plant disease and its severity, mycorrhizal colonization, and its strategies for overcoming plant immune defenses as well as suggesting how these interactions could be harnessed to increase plant productivity and control of phytopathogens.

## Overview of plant immune defenses

Defense is life. Every terrestrial plant is endowed genetically with genes whose products are responsible for protecting the plants from invasion by pathogens. Various microbes possess extracellular cell wall components, which are unique and conserved among microbes. These cellular structures are chitin (specific components of fungi cell wall), flagellin, lipopolysaccharides, and peptidoglycan (are major components of bacterial cell wall), EF-Tu (bacterial elongation factor) as well as β-glucans (peculiar to oomycetes) or glycoproteins (for viruses). These cellular structures are known as elicitor or microbe-associated molecular pattern. Pathogens and non-pathogenic organisms possess these elicitors. They are recognized, and their patterns are decoded by plant receptors known as pattern recognition receptors. Once in contact with the plant receptors, an immune response is triggered in the plant to adjust its physiological state from growth to defense. This switch of physiological status is geared toward aiding the plants to ward off pathogens or suppressing their proliferation at the site of infection (Jones and Dangl, 2006).

**Figure 1:**
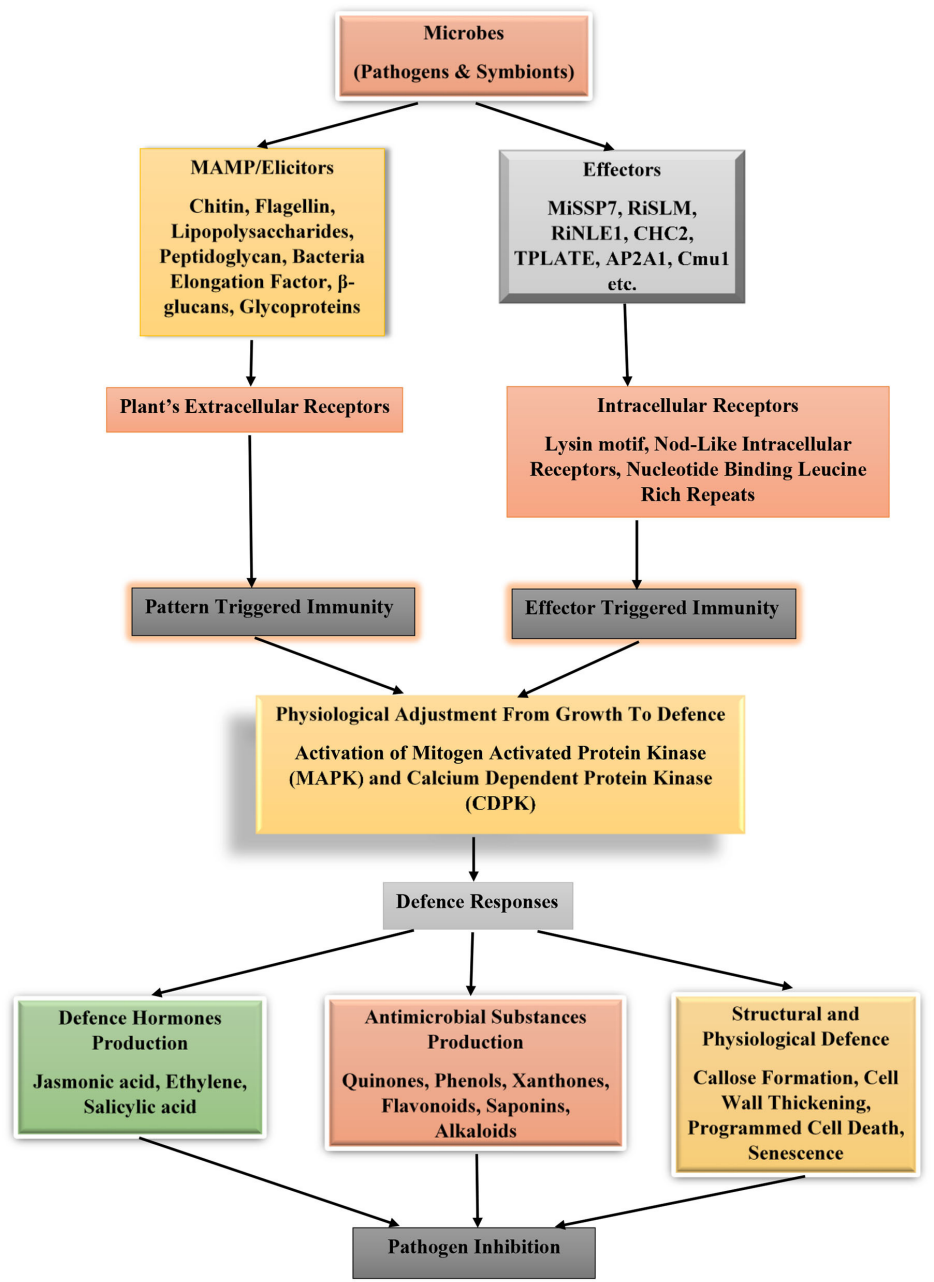
Plant immune system activation under the influence of effectors and microbes associated molecular patterns from microbes. MiSSP7 (mycorrhizal-induced small-secreted protein 7), RiSLM (a lysin motif (LysM) effector), RiNLE1 (nuclear localized effector 1), CHC2 (clathrin heavy chain 2), TPLATE, and AP2A1 (adaptor-related protein complex 2 alpha 1 subunit), and Cmu1 (chorismate mutase).

The underlying mechanism involving the activation of plant immunity by microbes is through defense pathways involving mitogen-activated protein kinase (MAPK) and Ca^2+^-dependent protein kinase (CDPK). These activated pathways will stimulate the production of ethylene (a stress hormone) and increase calcium ion flux and production of reactive oxygen species (Boutrot et al., 2010; Berens et al., 2017; Kourelis and Van Der Hoorn, 2018) that counteract the invader's activities (Figure 1). In addition to the cell surface receptors used by plants to detect pathogens in the environment, they equally possess intracellular receptors that detect protein molecules known as effectors. Effectors are chemical substances introduced into the plant cell upon infection by associated microbes (pathogens or symbionts), which perform various functions such as interference with the formation of immune protein complexes as observed in Jsi1 (jasmonate/ethylene signaling inducer 1) produced by the pathogen *Ustilago maydis*. This effector when bound to co-repressor proteins TPL/TPR (Topless and Topless-related proteins) will induce ethylene signaling and prevent the formation of ERF (ethylene response factor)-TPL/TPR complexes (Darino et al., 2019). Some microbial effectors possess enzymatic activity as Cmu1 (chorismate mutase), which diverts the pool of chorismate to produce phenylalanine and tyrosine through the shikimate pathway. Fungi infection of wheat by *P. striiformis f. sp. tritici* is facilitated by the interaction of effector Pst18363 with TaNUD23 (a wheat nudix hydrolase) to suppress the production and accumulation of reactive oxygen species in the infected plants (Djamei et al., 2011; Yang et al., 2020), in general effector functions as inhibitors or enzyme activity modulators in the host plants. A typical example of inhibitor effector is VdSCP41 that interferes with TFs (calmodulin-binding transcription factors) CBP60g and SARD1 from induction of defense genes in plants (Qin et al., 2018).

Effectors aid microbes to counteract or block pattern-triggered immunity induced by microbe-associated molecular pattern, thus enhancing colonization and infection. To detect these effectors, plants use NLRs (Nod-like intracellular receptors or nucleotide-binding leucine-rich repeat) to pick a change in host protein conformation or through NLR direct interaction with the effector molecules. NLRs act as sensors of microbial effectors. They are classified into three categories, namely TNL (Toll/interleukin1 receptor domain), CNL (Rx-type coiled coil), and RNL (RPW8-type coiled coil) (Jones and Dangl, 2006; Lee et al., 2019; Albert et al., 2020; Tamborski and Krasileva, 2020). This process of induction of plant immune response because of effector presence within the plant cells is effector-triggered immunity.

Another set of plant immune defenses is through structural barriers such as the presence of cell wall thickening, callose formation, and programmed cell death (Enebe and Babalola, 2019). These structural barriers prevent microbes from infecting and establishing themselves on a susceptible host. Some plants possess hydrolytic enzymes like myrosinase that break the bond between glucose and sulfur in glucosinolates to generate isothiocyanates, a toxic compound that affect pest. Leaves of cruciferous plants store large number of these enzymes. Other chemicals accumulated in plants are terpenoid, flavonoids, and phenylpropanoid (Oliver et al., 2009; Romani et al., 2020). Other useful chemicals produced by plants, which aid their resistance to phytopathogen attack, are salicylic, ethylene, and jasmonic acids (Enebe and Babalola, 2019; Steinbrenner, 2020; Fujita et al., 2022).

## Mycorrhizal fungi, colonization, and symbiosis

To repeat, mycorrhizal fungi are obligate biotrophic fungi, which depend on their host for carbon and in return aid the plant with nutrient and water uptake, tolerance to stresses (biotic and abiotic), and improve plants' growth and productivity. Their relationships with plants are mutualistic and beneficial to both the fungi and plants. This class of fungi equally mediates the interaction of plants with soil microbes, including mycorrhizosphere mutualists alike. These mycorrhizosphere mutualists provide plants with nitrogen through fixation, enhance nutrient mineralization, and produce vitamins and plant growth promotion hormones (Buee et al., 2009; Gomes et al., 2022). Through their actions, mycorrhizal fungi exert an enormous influence in driving the traits of plants below the soil surface as well as facilitating plant-to-plant communication through their hyphal interactions with plant roots (Simard et al., 2012; Werner et al., 2016; McCormack et al., 2017). In this process, nutrients, carbons, and signaling molecules can be exchanged between hetero- and conspecific plant species and between photosynthetic and non-photosynthetic plants. Exchange of nutrients among plants is highest among plants of the same species and increases the availability of nutrients (Argüello et al., 2016). Mycorrhizal fungi, through its mycelial networks, transport plants' photosynthates (carbon, sugars), polyols, and amino acids into the soil to sustain the biological activities of mycorrhizal-associated microbiomes (Buee et al., 2009). Additionally, non-host plants' growth and germination could be reduced by mycorrhizal fungi (especially ericoid and ectomycorrhizal fungi) through the production of allelochemicals and/or direct interactions (Rinaudo et al., 2010; Tedersoo et al., 2020). This fungi allelochemical effect is a strategy for promoting plant invasiveness and dominance in a particular environment. Mycorrhizal fungi could facilitate the introduction of plants into a new environment and aid their adaptation and survival (Kowalski et al., 2015). Surprisingly, arbuscular mycorrhizal fungi exhibit no known allelopathy in their association with plants (Wagg et al., 2015). In plant-to-plant communication, mycorrhizal fungi deliver signals of “warning” from pathogen-infected or pest-infested plants to healthy neighboring plants. The transfer of these signaling effects could occur through salicylic or jasmonic acid pathways to induce the expression of defense genes for the production of defense molecules that will deter or inhibit the invading pathogens and pests (Johnson and Gilbert, 2015; Song et al., 2015).

Mycorrhizal fungi, holistically, could be classified into four major types or groups based on their evolution, ecology, and morphology as ectomycorrhizal, arbuscular mycorrhiza, orchid mycorrhiza, and ericoid mycorrhiza. For details on their morphological differences, evolution, and ecology, readers should refer to Smith and Read (2008) and Tedersoo et al. (2020). Among these classes of mycorrhizal fungi, arbuscular mycorrhizal fungi (an endomycorrhizae) are the most ancient fungi, which form relationships with plant species that have non-multicellular roots like bryophytes, ferns, and lycophytes (Strullu-Derrien et al., 2018) as well as higher plants with multicellular root systems (MacLean et al., 2017). They associate with the roots of vascular plants by penetrating the root cortex (or cortical tissues) with their hyphae and forming arbuscules (site for nutrients exchange) and food storage hyphal swelling known as vesicles, hence the name vesicular-arbuscular mycorrhizal fungi. The mycorrhizal fungi belonging to this genera of endomycorrhiza are *Funneliformis, Glomus, Entrophospora, Gigaspora, Archaeospora, Acaulospora, Sclerocystis*, and *Scutellospora* (Balestrini et al., 2015). Endomycorrhizal fungus aids growing plants in water uptake and survival in water-stressed environment through direct regulation of plants' stomatal conductance (Augé et al., 2015). They play a prominent role in plant uptake of phosphorus and other nutrients from the soil (Smith et al., 2003). A study has shown that arbuscular mycorrhizal fungi association could trigger in roots the expression of genes responsible for phosphorous transport (StPT3 and StPT4 of potato) or uptake through the activation of production of signaling molecules (lysolipid lyso-phosphatidylcholine) (Drissner et al., 2007). This chemical in turn induces the activation of the phosphorus transporter that aids phosphate absorption. Plants generally take up nutrients (phosphorus) either through root absorption or mycorrhizal hyphal mediated uptake. Another group of mycorrhizal fungi based on their hyphal arrangements within the plants' cortical tissues are the ectomycorrhizal fungi. They belong to basidiomycetes and ascomycetes as well as form association with 10% of terrestrial plants, mostly trees. On the feeder roots, they form a fungal mantle. Unlike endomycorrhizal fungi, they penetrate the roots and form hartig net, modifying plant roots into swollen and branched hyphae.

As biotrophic fungi, mycorrhizal fungi must depend on their host for nutrition in exchange for nutrient-facilitated uptake and other ecological services provided to the plant. For this to happen, the fungi must colonize the plant and establish a symbiotic relationship. The process begins with the finding of the host root, which is crucial for the process of fungal root colonization. This is followed by biochemical sensing and responding to the stimuli communication between the host and the fungi and finally the fungus getting inside the host root to colonize it and complete the mycorrhizal association. During the pre-colonization phase, the plant secretes chemical signal molecules known as strigolactones. Strigolactones are carotenoid-based phytohormones that promote hyphal branching, development, and metabolism of the fungus. This carotenoid phytohormone was first discovered in *Petunia hybrid* plants and is exported out of the plant by a membrane-bound exporter “pleiotropic drug resistance 1” (PDR1) through suberin-free hypodermal passage cells (HPCs) found in the exodermis of the plant roots. This membrane-bound exporter (PDR1) has been found to contribute to the build-up of strigolactones in the rhizosphere environment (Akiyama et al., 2005; Kretzschmar et al., 2012; Sasse et al., 2015). In some plants such as *Medicago truncatula* ABCG59, which lack an apoplastic hydrophobic diffusion barrier, strigolactones produced within the plant root cells diffuse passively into the rhizosphere without the aid of specialized exporters or transport apparatus (Banasiak et al., 2020). Other chemically secreted substances by plant roots that facilitate hyphal tip elongation, growth, and branching are 2-hydroxyl fatty acids and flavonoids (Scervino et al., 2007; Banasiak et al., 2021).

**Figure 2:**
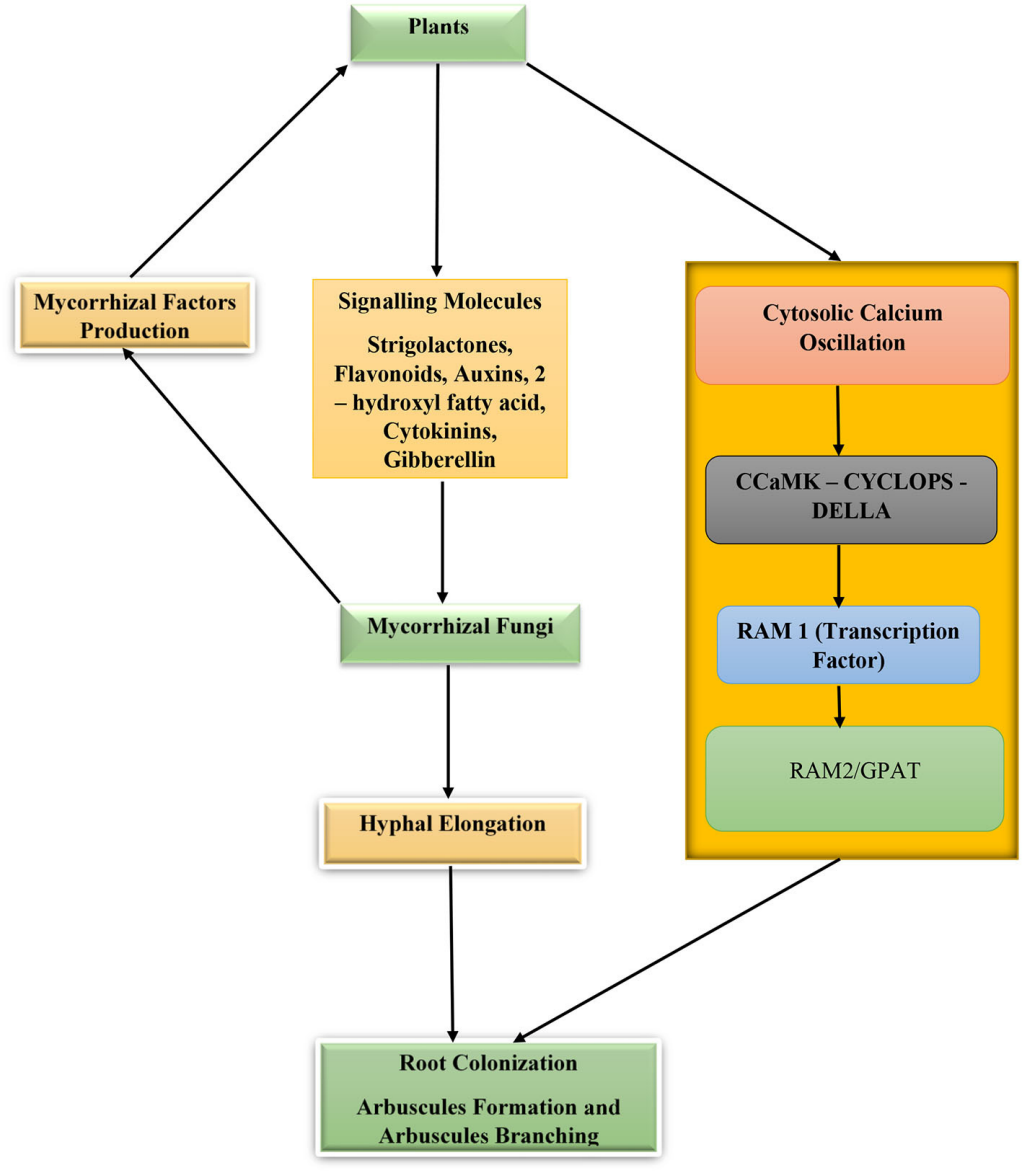
Plant–mycorrhizal fungi interactions for root colonization and symbiosis. Calmodulin-dependent protein kinase (CCaMK), CYCLOPS (a SYM genes product and a transcription factor), required for arbuscular mycorrhization 2/glycerol-3-phosphate acyl-transferase proteins (RAM2/GPAT), and required for arbuscular mycorrhization 1(RAM1).

In response to these signal molecules, the mycorrhizal fungi progress with growth toward the source of the signal and secrete a group of chemical substances collectively called mycorrhizal factors (Myc) (chitooligosaccharides and lipo-chitooligosaccharides). These mycorrhizal factors are recognized by the plants' Myc factor receptors and SYMPK (a receptor like kinase) that through phosphorylation activities transmit signals from the cytoplasm to the plant nucleus. During the process of myc factor receptor detection of myc signal, the plant root cells secrete cytosolic calcium whose concentrations alternate repeatedly in the cytoplasm and nucleus. As the calcium concentration oscillates, the concentration gradients are decoded by a protein kinase—calmodulin-dependent protein kinase (CCaMK) whose activity and phosphorylation of CYCLOPS (a SYM genes product and a transcription factor) will result in enhanced root colonization by the fungi (Parniske, 2008; Maillet et al., 2011; Gutjahr and Parniske, 2013; MacLean et al., 2017; Mohammad, 2019; Semchenko et al., 2022). In the case of arbuscular mycorrhizal fungi, the fungal hyphae digest the tissue of the plant roots and enter the cortical cells to produce clusters of divided hyphae that form arbuscules. Required for arbuscular mycorrhization/glycerol-3-phosphate acyl-transferase proteins (RAM2/GPAT) are essential for arbuscule development and root surface hyphopodium formation (Figure 2). The hypodermal passage cells influence the penetration of mycorrhizal fungus hyphae into the host roots and the excretion of strigolactones. The higher the number in the roots, the higher the root colonization by the fungi, although their distribution and number are controlled by plant genotype, environmental factors, and hormones (Liu et al., 2019; Banasiak et al., 2021). Additionally, chemical substances such as phytohormones (cytokinins, auxins, ethylene, strigolactones, and gibberellins) are key hormones regulating mycorrhizal development, root colonization, and symbiosis (Bedini et al., 2018).

One may wonder why mycorrhizal fungi, despite its biotrophic lifestyle, could successfully establish a “permanent” association with host plants. This issue will form the basis of our discussion in the following section.

## Mycorrhizal strategies for suppressing plant immune systems and enhancing their establishment on host plants

Recall, plants are equipped with immunological tools to ward off, destroy, and control the invasion of pathogenic microbes, which may seek to attack these plants. During contact with foreign chemical signatures that degrade plant cell wall or in contact with microbes, an innate immune signal network is activated. Microbes possess cell wall/membrane structures that are conserved among them (such as peptidoglycan, flagellin, lipopolysaccharides, and chitins). These cellular structures are known as microbe-associated molecular pattern and are recognized by the plant receptors also known as pattern recognition receptors. The contact of these two cellular structures will automatically trigger an immune response in plants in a phenomenon known as pattern-triggered immunity. In some cases, the microbes might succeed bypassing recognition by the plants and inject chemical molecules (effectors) which are further detected by intracellular receptors. These intracellular receptors activate the plant immune response known as effector-triggered immunity. In response, the plants could either produce defense phytohormones (jasmonic acid, ethylene, and salicylic acid) or antimicrobial substances (quinones, phenols, xanthones, flavonoids saponins, and alkaloids), which participate in the inhibition of the microbial growth, replication, and activities. Some structural and physiological defense effects will be exerted on the infected cells, such as callose deposition, cell wall thickening, programmed cell death, and senescence etcetera (Ramirez-Prado et al., 2018; Enebe and Babalola, 2019; Favre-Godal et al., 2020). Both biotrophic and necrotrophic microbes (pathogens and non-pathogens alike) trigger plant immune response. Salicylic acid is the predominant phytohormone defense chemical peculiar with plant response to the control of biotrophic microbes, while necrotrophic ones trigger jasmonic and ethylene defense hormones in plants (Berens et al., 2017; Xu et al., 2018).

Therefore, since mycorrhizal fungi exhibit biotrophism and contain beta glucan and chitin in their cell walls (Bartnicki-Garcia, 1987; Zeng et al., 2020), they certainly have no exception in triggering plant immune responses during infection, colonization, and symbiosis. Studies have shown that during the initial phase of mycorrhizal fungi interaction with plants, an increase in chitinase activity in the plants was detected (Spanu et al., 1989; Volpin et al., 1994). This shows the activation of the plant immune response; however, as the interaction progress, the enzyme activity of chitinase and other defense chemicals decreased or became completely repressed (Spanu et al., 1989; Gianinazzi-Pearson et al., 1996). In another study, pathogenesis-related genes were transiently induced in plant (barley) roots at the later stage of plant–fungi interactions, resulting in the suppression of the plant defense ability in the presence of mycorrhizal fungi association (Deshmukh and Kogel, 2007; Schäfer et al., 2009; Plett and Martin, 2018). Plant immune responses are triggered; however, the fungi explore different strategies for suppressing plant immunity. These immune-suppressing strategies could be categorized as direct and indirect approaches. The direct suppression approaches are as follows: (1) production and injection of effectors, (2) enhancement of root cortex cell division, (3) scavenging of reactive oxygen species, and (4) recruitment of jasmonic acid and/or gibberellin signaling pathway, while the indirect strategies are cellular-structural reprogramming and/or transcriptional regulation and plant intrinsic properties modification.

## Effector approach

To establish mutualistic relationships and particularly adapt to the lifestyle of obligate biotrophism, mycorrhizal fungi have evolved unique strategies for evading or downregulating plant immune response. Upon infection of a susceptible host, mycorrhizal fungi produce and inject protein molecules, which act as negative regulators of plant defense genes. These negative regulatory activities shut down the production of defense chemicals such as salicylic acid, jasmonic acid, and/or ethylene. These fungi-secreted proteins are called effectors. Different mycorrhizal fungi induce different effectors. For instance, *Laccaria bicolor* (an ectomycorrhizal fungi) secretes MiSSP7 (mycorrhizal-induced small-secreted protein 7) that interact with repressor proteins for jasmonic acid inducible genes and results in the repression or downregulation of the defense constitutive genes for jasmonic acid production, thereby enhancing plant–mycorrhizal fungus interactions. The production of this effector protein by the fungi is activated in response to plant secretion of quercetin and/or flavonoid rutin. These exudate compounds activate the fungi biological processes that lead to the expression of effectors and establishment of mutualistic relationships (Plett et al., 2011; Favre-Godal et al., 2020; He et al., 2020). In another study, endomycorrhizal fungi (arbuscular mycorrhizal fungi—*Rhizophagus irregularis*) in an interaction with Medicago plants produced RiSLM (a lysin motif (LysM) effector) in its intraradical mycelium. This effector protein functions by binding to chitin oligosaccharides on the fungi cell walls, thereby protecting them from the hydrolytic action of plants produced chitinases. RiSLM effector aids the subverting of chitin-triggered immune responses such as induction of defense gene expression or the production of reactive oxygen species. Silencing of genes responsible for the production of RiSLM effector or the plant lysin motif plasma membrane receptors could lead to a reduction in fungi colonization of the host plant (Bozsoki et al., 2017; Zeng et al., 2020). Effector such as RiSLM, despite its affinity to the lipo-chitooligosaccharides, chitosan, and chitin or Myc factors, they do not interfere with signaling and symbiotic interaction between the fungi and its host (Maillet et al., 2011; Zeng et al., 2020). The expression of this effector by the fungi is under the influence of plant-secreted strigolactones, which activate its production during the establishment of plant–fungi symbiosis. Germinating spores of mycorrhizal fungi treated with rice root exudate containing strigolactone and/or strigolactone analog (GR24) show the expression of lysin motif effector (RiSLM) by the fungi and further justify its possible role in enhancing fungi infection, colonization, and symbiosis with the host plants (Tsuzuki et al., 2016; Nadal et al., 2017).

Interestingly, the RiSLM effector acts both within and outside the plant roots during the mycorrhizal fungus infection, and RiNLE1 (nuclear localized effector 1) acts intracellularly. RiNLE1 effectors are produced by *R. irregularis'* arbuscules within the infected plant tissues. This effector migrates to the host nucleus, interacts with H2B (plant core nucleosome protein histone 2B), and impairs the physiological functions of mono-ubiquitination of H2B. The impairment of nucleosome protein histone 2B by the RiNLE1 effector leads to the suppression in the expression of host defense-related genes and enhancement of fungi–root colonization (Wang et al., 2021). Rationally, it can be easy to understand the interactions and migration of RiSLM effectors with plant receptors and fungi chitin during immune subverting exercise, but it remains unclear the translocation approach of the RiNLE1 effector into the plant nucleus and requires further studies. It has been observed that transcriptional activation of resistance genes of Arabidopsis plants in the presence of necrotrophic pathogens requires the action of H2B mono-ubiquitination (Zou et al., 2014), and its impairment would result in increased colonization and infection of the plants. One major function of the RiNLE1 effector is the modification of histone 2B proteins and blockage of HUB1 (RING E3 ligase H2B monoubiquitination1) enzyme accessibility to histone (mono-ubiquitinate H2B). This sequence of events involving the interactions of effector proteins and histone molecules is a form of epigenomic reprogramming employed by mycorrhizal fungi during symbiosis with plants. Another effector protein produced by *R. irregularis* is the SP7 protein, which interacts with transcriptional factors within the nucleus and prevents the expression of plant defense genes, thereby enhancing fungi colonization (Kloppholz et al., 2011).

While some effectors aid the suppression of plant expression of pathogenesis-related genes or defense genes, others like CHC2 (clathrin heavy chain 2), TPLATE, and AP2A1 (adaptor-related protein complex 2 alpha 1 subunit) are endocytic effectors with cell division activation mechanisms. They promote root cortex cell division and multiplication during fungi colonization, resulting in enhanced accommodation of the mycorrhizal fungi within the plant tissues. These effector proteins are upregulated in the plant-infected cells during fungus accommodation (Russo et al., 2019).

## Deacetylation of chitin

Fungi cell walls are composed of chitin. Chitin is a polymer composed of unbranched beta 1,4 linked N-acetylglucosamine molecules and when broken down generate chitooligosaccharides. Both chitin and its breakdown products are recognized by plant receptors (lysin motif plasma membrane receptors) that trigger plant immunity. Chitin could best be described in the context of plant immune activation as microbe-associated molecular pattern (Zeng et al., 2020), which induce pattern-triggered immunity in plants. Zeng et al. (2018) reported that extraradical mycelia of mycorrhizal fungi produce chitin deacetylases enzyme which participate in chitin deacetylation more outside the host plant, but the genes producing these enzymes are suppressed or downregulated when the fungi are growing within the plant tissues intraradically. Hence, as a survival strategy, mycorrhizal fungi express mostly acetylated chitin within the host tissues. Suffice to say, deacetylation process converts chitin to chitosan and plant receptors do not recognize deacetylated chitosan and so no pattern-triggered immunity is elicited (Liu et al., 2012).

## Recruitment of jasmonic acid and/or gibberellin signaling pathways

Every biotrophic microbe undergoing biotrophic growth in plants will be confronted with a functional and active root immune system. Mycorrhizal fungi (*Piriformospora indica*) growing on *Arabidopsis thaliana* roots are no exception. These fungi although beneficial to plants do not evade detection by plant immune surveillance and receptors. One may wonder how these fungi bypass the host immune response. The answer lies in its ability to suppress plant immune response through the recruitment of jasmonic acid and/or gibberellin signaling pathway. *P. indica* colonization of plants was proposed to exhibit different degrees of colonization at different stages, such as extracellular colonization of root surfaces, biotrophic colonization (rhizodermal and cortex cells colonization), cell death colonization, and reproduction phase (Jacobs et al., 2011). Biotrophic colonization involves hyphal penetration and growth into the cell through plasma membrane invagination without lysis of the cytosol or tonoplast structure. The reverse happens at the cell death colonization phase. Nevertheless, plant seedlings, infected with mycorrhizal fungi and under treatment with flagellin (flg22) or fungi chitin, suppress the plant immune system, callose deposition in both the colonized area and the entire elongation, and/or differentiation zones of the plant roots. It prevents flg22- and elf18-induced growth inhibition. To perform these immune suppression activities, jasmonic acid signaling pathways are recruited by the fungi since jasmonic acid has affected the actions of glucosinolate-associated defense and salicylic acid (Jacobs et al., 2011). Jasmonic acid is a C12 cyclopentanone fatty acid that is required for the activation of arbuscular mycorrhization 2 (RAM2) (a gene encoding glycerol-3-phosphate acyl transferase) whose signal is required for the mycorrhizal fungus penetration of host plants (Gobbato et al., 2012; Liu et al., 2022). Mycorrhizal fungi dependence on jasmonic acid signaling for its colonization and suppression of plant immunity is a function of jasmonic acid counterbalancing effect on salicyclic acid. Salicylic acid, a major defense-related hormone, is responsible for controlling biotrophic microorganisms. Necrotrophic microorganisms are controlled by jasmonic acid and ethylene (Berens et al., 2017; Xu et al., 2018). Hence, mycorrhizal fungi exhibit an obligate lifestyle of biotrophism that requires jasmonic acid for its successful colonization and suppression of plant immunity. Another phytohormone that facilitates root colonization by mycorrhizal fungi is gibberellin. A study has shown that mutant plants defective in gibberellin synthesis reduce root colonization, as observed in Arabidopsis and barley roots with *P. indica* (Schäfer et al., 2009). Similar observations were reported by Jacobs et al. (2011).

## Loss of physiological functions

For a relation to be considered mutual, it will be based on reciprocity. Endomycorrhizal fungi have evolved a unique strategy of partial or complete loss of fatty acid biosynthesis enzyme and depend entirely on the host plants to meet their fatty acid need, through the actions of required for arbuscular mycorrhization 1 (a transcriptional factor), which regulates symbiosis-induced genes (WRI5a, STR, FatM, and RAM2) that facilitate plant production of lipid, its accumulation, and transfer from the plant host to the fungi (Jiang et al., 2018; Müller et al., 2020). Also lost is the mycorrhizal fungus synthesis of cell wall degrading enzymes (such as cellulase and pectinase) during biotrophic growth within the plant tissue (Wan et al., 2018; Delaux and Schornack, 2021) without lysis of the cytosol or tonoplast. Only extracytosolic growth (i.e., invagination of the plasma membrane) is observed. Functions involving nutrient uptake by the fungi and transfer to the host plant are maximized in exchange for the photosynthates. The loss of functions that ensure complementarity is the driving force that sustains the symbiotic interactions. This feature is peculiar in symbionts but absent in pathogenic microbes whose virulence and activities lead to disease and death of their host.

## Cellular reprogramming, regulation, and plant genetic make-up

The development of mycorrhizal symbiosis and plant accommodation of the fungus arbuscules require plants transcriptional control and reprogramming of cellular activities. This process leads to morphological and cellular changes in the arbuscule harboring root cells. For arbuscules to form, certain transcriptional changes in genes regulating mycorrhization will occur (Pimprikar and Gutjahr, 2018). Recall that gibberellin is an essential hormone required for efficient root colonization by mycorrhizal fungi (Schäfer et al., 2009) and is under the control of DELLA proteins (Silverstone et al., 1998). When CYCLOPS (DNA-binding transcription factor) and CCaMK (calcium calmodulin-dependent kinase) interact with DELLA proteins in the presence of signals or effectors from the fungi, RAM1 is activated and arbuscules are developed. However, the interaction of DELLA protein with gibberellin is antagonistic and subsequently leads to DELLA protein degradation when it binds to the gibberellin receptors, thus inhibiting the development of arbuscules and activation of RAM1 (required for arbuscular mycorrhization 1) (Tirichine et al., 2006; Singh et al., 2014; Pimprikar et al., 2016; Gong et al., 2022). DELLA proteins also possess anti-mycorrhization effects such as facilitating the degradation of arbuscules through the activation of genes responsible for the production of chitinase and proteases (Floss et al., 2017; Ho-Plágaro et al., 2021). Such chitinase producing gene is class 3 chitinase (Mtchitinase III-3) which is expressed in mycorrhizal fungi-colonized root cells. GRAS transcriptional factors produced by GRAS genes (gibberellin-acid insensitive (GAI), a repressor of GA1 (RGA), and scarecrow-like (SCL) proteins) are important in the regulation of biological processes in plants, especially during the process of mycorrhizal symbiosis (Pysh et al., 1999). Ho-Plágaro et al. (2019) reported an increase in GRAS genes (SlGRAS18 and SlGRAS43) in arbuscules containing tomato root cells. Silencing of these genes (SlGRAS18 and SlGRAS38) resulted in delayed mycorrhization, which could be explained by the degradative effects of DELLA proteins on arbuscules. Therefore, the formation of a symbiotic relationship between plants and mycorrhizal fungi involves not only cell wall modification and signal transduction but also nutrient transport protein activation. For instance, the exchange of phosphorus between the fungi and the host plant depends on hydrogen ion gradients produced by hydrogen ion-ATPase domicile on the peri-arbuscular membrane. This peri-arbuscular membrane, ATPase apparatus (SlHA8) is required for nutrient exchange and arbuscule development. Silencing or deletion of this gene has been shown to negatively affect plant nitrogen and phosphorous contents of the plants as well as the structure of arbuscules, suggesting the important functions of SlHA8 in nutrient uptake and mycorrhizal symbiosis in plants (Liu et al., 2021). Another example is recorded by Liu et al. (2022) who observed that rice plants defective in OsRAM2 significantly affect arbuscule formation and mycorrhizal colonization. This gene is involved in the transfer of lipids from plant to the fungi as well as in signaling and is expressed mostly in cells harboring arbuscules. A similar result is recorded in the study of Dai et al. (2022).

Additionally, auxin (indole-3-acetic acid—IAA) production and accumulation in the plant promote mycorrhizal symbiosis. Induction of the GH3 gene (SlGH3.4 that encodes IAA-amido synthetase) is responsible for the regulation and cellular homeostasis of auxin that has a direct effect on arbuscule development. In mycorrhized roots, auxin-induced expansin genes are upregulated to ensure the maintenance of auxin concentration and efficient development of arbuscules in the root cortex (Chen et al., 2022). Excessive presence of auxin inhibits arbuscule development and incidence. Plants perhaps control the expression of the genes for adequate establishment of symbiosis with mycorrhizal fungi. Mycorrhizal symbiosis, on the contrary, has a modulatory effect on the plant lateral root development through its controlling impact on the CEP2 (C-terminally encoded peptide) expression and auxin-related pathway. Critical to the lateral root formation is the presence of CEP2, which is downregulated during the process of mycorrhizal symbiosis to curb the negative effect of CEP2 on lateral root formation (Hsieh et al., 2022).

Other genes essential for the development of mycorrhizal fungi colonization are a kinase known as arbuscule development kinase 1 (OsADK1) which is induced in cortical cells containing arbuscules. Mutation of this gene was found to lower *R. irregularis* colonization of rice roots as it has an effect on the activities of RAM1 and WRI5 transcriptional factors (Guo et al., 2022). RAM1 is a transcription factor which is involved with mycorrhization and acts upstream of the gene—KIN3 as well as RAM2. The expression of KIN3 results in the suppression of plant defense-related genes in response to plant–fungi symbiosis (Gobbato et al., 2012; Irving et al., 2022). Reprogramming and modulation of these genes ensure the sustenance of the association between the mycorrhizal fungi and its host. Studies should focus on detailed understanding of the signal transduction channels that determine the tightly controlled plant–mycorrhizal fungi interactions and how plants evolve to differentiate symbionts from pathogens in its response to cellular modification and transcriptional reprogramming in the infected cells.

## Plant immune suppression—the consequences of mycorrhizal colonization on disease incidence and pest infestation

**Figure 3:**
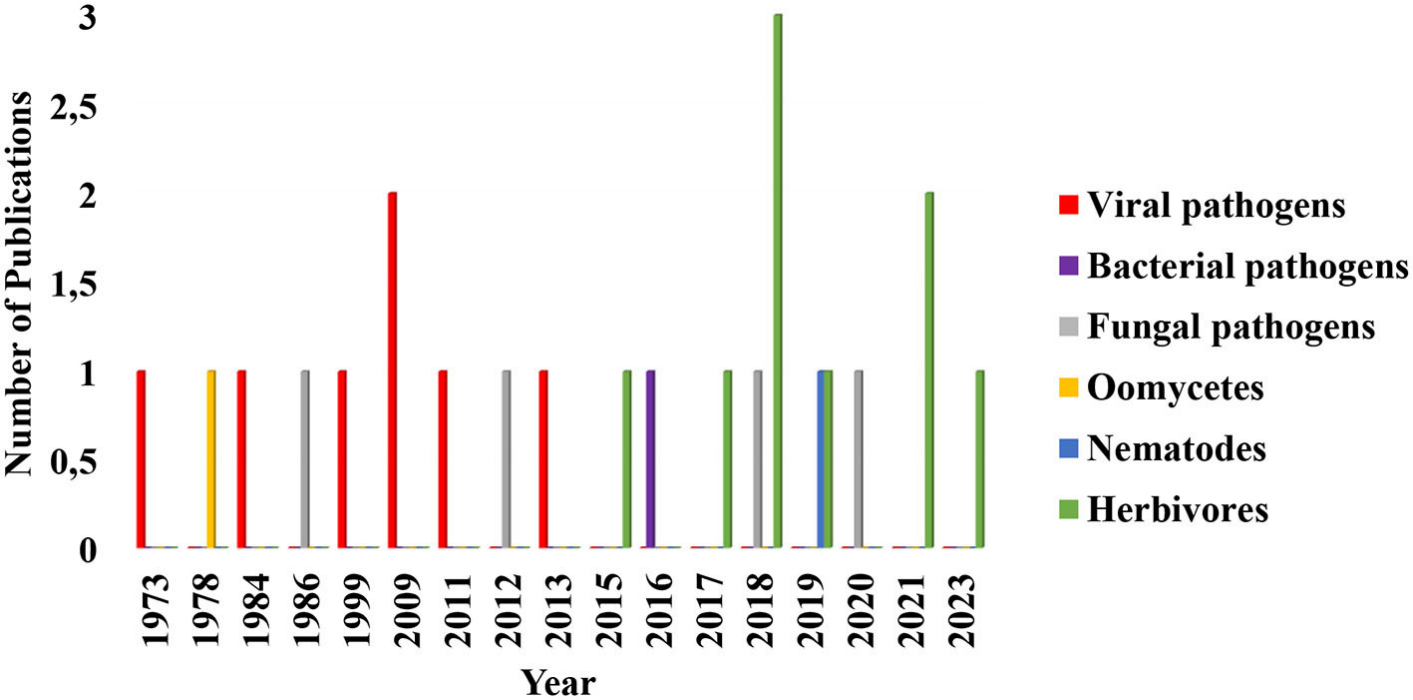
Diagrammatic representation of the trend in research and publication on the role of arbuscular mycorrhizal fungi in the enhancement of plants susceptibility to phytopathogens and herbivores in the last 50 years. Each search was performed on Google scholar, Jstor, and Sciencedirect. Research up until now has focused more on mycorrhizal fungal induction of plants resistance to phytopathogens and herbivores as well as on host plant level research and its ecological roles. In this diagram, publications in arbuscular mycorrhizal fungal induction of plants susceptibility to viral pathogens remained the same from 1973 to 2013 with exception of 2009 which is slightly high. Those on herbivores remained consistent from 2015 to 2023 except for 2018 and 2021 that are higher in numbers. Oomycetes, bacterial and fungal pathogens, remained the same across the years. Publications selected and included in this chart fulfilled this criterion: it must quantitatively show how arbuscular mycorrhizal fungal colonization of the plants increased the incidence of the disease and herbivores population densities and survival. The data used in plotting this figure is contained in Supplementary Table S1.

Several studies have focused on the efficacy of arbuscular mycorrhizal fungi contributions to the biocontrol of plant pathogens such as nematodes, viruses, and bacteria through induction of plant immunity and activation of ethylene production (Campos-Soriano et al., 2012; Duc and Posta, 2018; Miozzi et al., 2019; Poveda et al., 2020). However, the consequences of these fungi–plant interactions on plant susceptibility to pests and pathogens are an area that seems to be receiving less attention (Figure 3).

Suffice to say, despite the numerous contributions of mycorrhizal fungi-induced plant resistance to pathogens, one would expect that commercially available biocontrol products marketed around the world would have mycorrhizal fungi as one of its active inoculants or ingredients. A typical biocontrol product contains either bacteria (from *Pseudomonas, Burkholderia*, and *Bacillus* species) or fungi (from *Gliocladium* and *Trichoderma* species) or a combination of both fungi and bacteria. Some biocontrol products may contain phages as the active inoculants (Whipps et al., 1988; Whipps and Davies, 2000). For instance, a commercial biocontrol product named intercept used for controlling the diseases caused by *Fusarium, Rhizoctonia solani*, and *Pythium* sp on cereal and vegetable crops contains *Pseudomonas* sp or *Burkholderia cepacia* as its biocontrol agents. Another product of fungi inoculants is PreStop, which is used in the control of diseases caused by *Rhizoctonia sp., Pytlzium sp., Didymella sp., and Botrytis sp.*, on vegetable and ornamental plants. A phage-based product is phages, which is used for the control of diseases caused by *Pseudomonas tolaasu* on mushrooms. However, none of these products contain mycorrhizal fungi (Whipps and Davies, 2000; Whipps, 2004). The question is why are mycorrhizal fungi absent in these commercially available biocontrol products?

**Figure 4:**
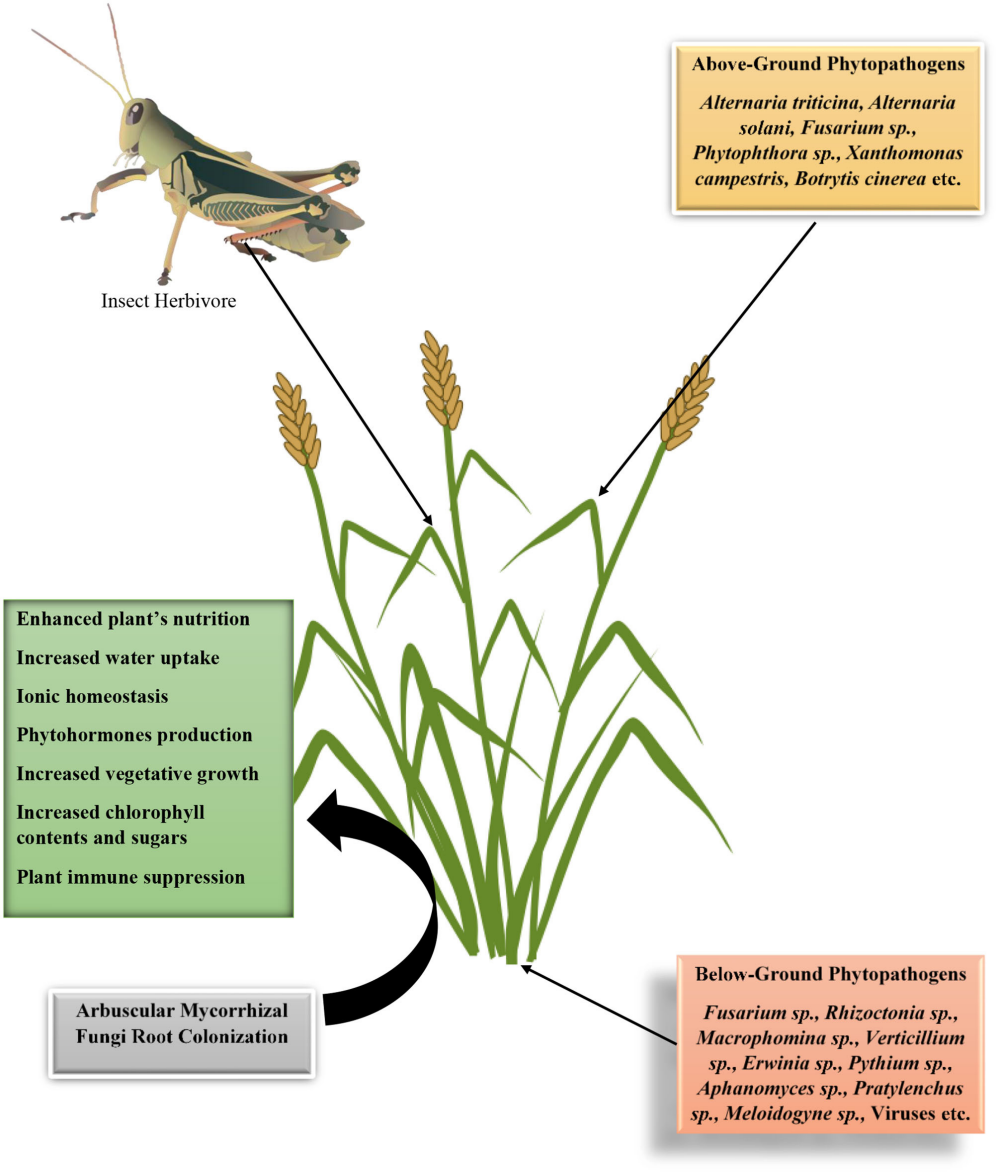
Diagrammatic illustration on the interactions of arbuscular mycorrhizal fungi in enhancing plants susceptibility to phytopathogens and insect herbivore based on its effects on plants' vegetative growth properties, physiology, and immune suppression.

In nature, every action, interaction, or relationship has corresponding reactions, consequences, drawbacks, or merits. Plant–mycorrhizal fungus interactions are no exception. It equally comes with a corresponding demerit in enhancing plant susceptibility to pathogens and pests. It has been proposed by Miozzi et al. (2019) that mycorrhizal fungi induce susceptibility in plants to viral infection. Some reasons have been put forward why mycorrhizal fungi that is beneficial to plants will have a corresponding negative effect on the development and incidence of diseases in plants. These are (1) increase in food nutritional quality, (2) increase in plant nutritional quantity, (3) suppression of plant immunity to establish symbiosis, and (4) mycobiome and mycorrhizal fungi microbiome effects. The first and the second hypotheses have been put forward by Bennett et al. (2006) to explain that increase in plant nutritional quality such as the phosphorus, nitrogen, and potassium contents will increase the available nutrients status for the pathogen or pests as well as the quantity of biomass available for meeting the nutritional needs of these organisms (Figure 4). In the previous section, we have described the various strategies mycorrhizal fungi employ in lowering plant immune systems, such as injection of effectors and deacetylation of chitin. The effects of mycobiome or mycorrhizal fungi microbiome in the transmission of pathogenic organisms from the soil into the plant tissue are yet be fully understood; however, we propose that this might be a probable mechanism through the enhancement of plant susceptibility in the presence of mycorrhizal fungi. We will now present evidence of mycorrhizal fungi induction of plant susceptibility to pathogens.

**Figure 5:**
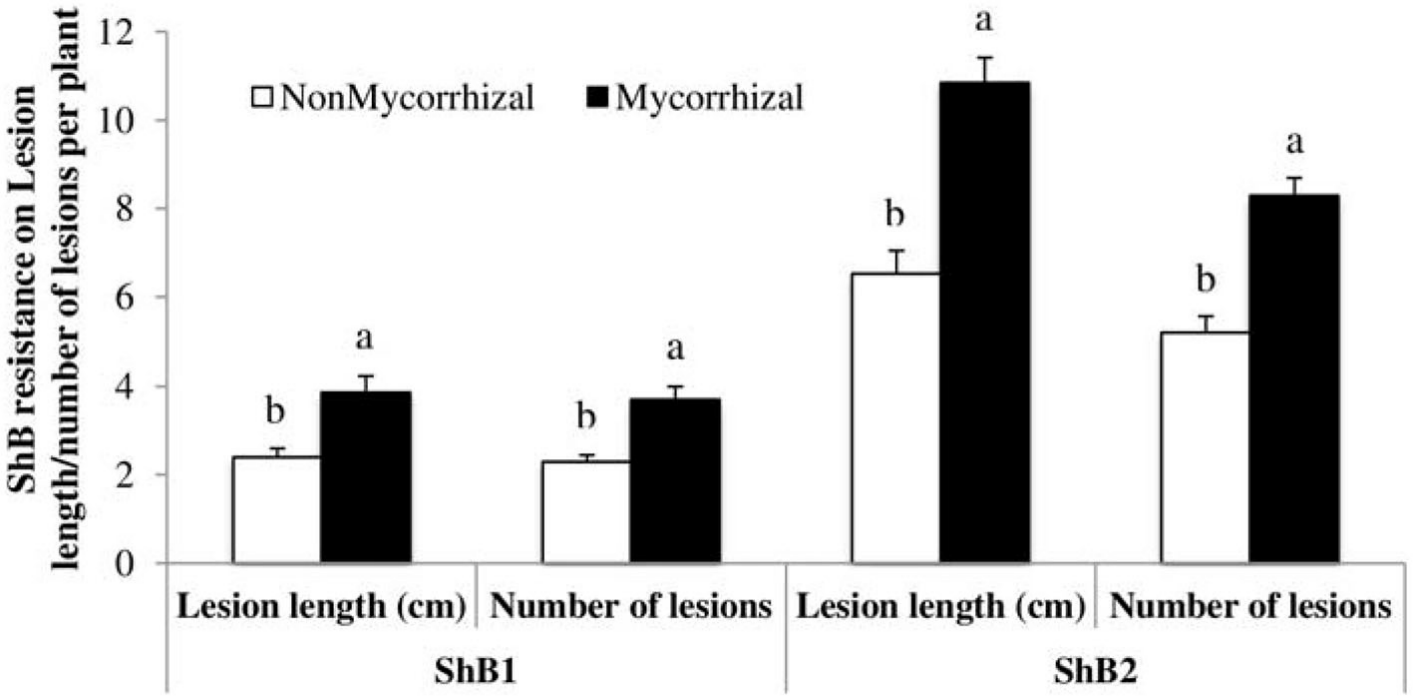
Case study on rice sheath blight disease variables (lesion length and number of lesions) measured after inoculation with isolate LR172 of *Rhizoctonia solani* in mycorrhizal and non-mycorrhizal rice plants in greenhouse experiments in the summer 2013. Non-mycorrhizal: rice seeds + sterilized AMF; mycorrhizal: rice seeds + live AMF. Bars and lower case letters at the column head indicate that means differ significantly (LSD, *P* ≤ 0.05). Adapted from Bernaola et al. (2018) published by Frontiers in Plant Science in 2018 with CC BY license permission.

Plants are challenged by several stressors ranging from pathogenic microbes to pests that depend on plants for nutrition and reproduction. Mycorrhizal fungi perform many beneficial roles such as promoting root hair growth, auxin synthesis, plant nutrients, and water uptake as well as increasing crop yield (Gosling et al., 2006; Liu et al., 2018), but its actions are not entirely beneficial as some could have cost implications to farmers such as induction of plant susceptibility to pathogens and pest, an increase in plant nutritional contents, and root and shoot growth that may increase the attraction level of the plants to soil-borne pathogens. Association of rice plants with mycorrhizal fungi has increased the susceptibility of the plant to *Rhizoctonia solani*, causing sheath blight in both greenhouse and field experimental settings. Susceptibility to sheath blight infection and lesion lengths observed were higher in mycorrhizal fungi inoculated plants. Further analysis showed that there was no significant difference in the plant nutritional quality between inoculated and uninoculated plants. This suggests that the main mechanism employed by the fungi in increasing plant susceptibility to the pathogen could be the suppression of plant defense arsenals (Bernaola et al., 2018) (Figure 5).

A typical example is mycorrhizal fungi altering of jasmonic acid pathway in tomato plants (Jung et al., 2012). In another study, co-inoculation of mycorrhizal fungi (*Glomus* species) with a pathogen fungi (*Verticillium dahliae*) at 20 mg per kg of phosphate exhibited severe wilting disease caused by the *Verticillium* pathogen compared with non-mycorrhizal inoculated cotton plants (Patale and Shinde, 2012), although this study was conducted under soil fertilization regime of 20 and 300 mg per kg of superphosphate fertilizer. At 300 mg kg^−1^ P, disease severity was observed in both mycorrhizal and non-mycorrhizal fungi-treated plants, implying that soil nutrients have an effect in increasing plant disease susceptibility due to their negative effect on mycorrhizal fungi colonization and a positive effect in increasing pathogen nutrition. This is supported by the Bennett et al. (2006) hypothesis as presented above. Other ideas that could support the observed disease severity as presented by Patale and Shinde (2012) include increase in plant root penetration by the pathogen enhanced by the mycorrhizal fungi colonization, plant potassium content dilution, and enhanced movement of pathogens' microconidia in plant tissue due to mycorrhizal fungi influence on transpiration and nutrient acquisition.

Surprisingly, the susceptibility effects of mycorrhizal fungi on colonized plants infected by the pathogens could be time-dependent. Inoculation of tomato plants with arbuscular mycorrhizal fungi (*Glomus mosseae*) and tomato spotted wilt virus did not show any immediate disease severity difference between mycorrhizal fungi inoculated and uninoculated control. However, as the time of interaction increased, the viral load and symptoms increased and recovery delayed in the infected mycorrhizal plants compared with the control. This shows that plant sensitivity to viral infection in the presence of mycorrhizal fungi is time-dependent (Miozzi et al., 2011). This is supported by the notion that the disease control potential of mycorrhizal fungi against pathogens in inoculated plants is dependent on time interval between mycorrhizal inoculations, colonization, and exposure of the colonized plants to the pathogens. For instance, pre-inoculation of peas with *Glomus fasdculatum* before exposing the inoculated plants to a root rot pathogen (*Aphanomyces euteiches*) improved plant resistance to the pathogen, but when the mycorrhizal fungi and the root rot pathogen are coinoculated on the plants, disease severity is observed and the mycorrhizal fungi could not suppress the pathogen (Bärtschi et al., 1981; Rosendahl, 1985), hence increasing its susceptibility to the pathogens.

Mycorrhizal fungi (*R. clarus* and/or *Claroideoglomus etunicatum*) inoculation of lemon grass (*Cymbopogon citratus*) challenged with pathogenic nematodes (*Pratylenchus brachyurus*) increased the nematode population in the infected plants by a magnitude of 8.6 more than the non-inoculated control without improving plant growth, but it improved the expression of defense-related enzymes (peroxidase, polyphenol oxidase, and β-1,3-glucanase) (e Silva et al., 2021). The increase in nematodes population could be a result of plant nutritional quality and quantity increased, which enhance their nutritional status and multiplication rate, while no obvious increase in the growth of the plants could be a metabolic energy switch to defense-related gene expression. Nematode and mycorrhizal fungi production of effectors could enhance plant susceptibility to the pathogenic nematodes (Grossi-de-Sa et al., 2019; Wang et al., 2021). Nematodes are equipped with physiological ability to feed on plant tissues and cause lesions and reproduce within the host plants faster than mycorrhizal fungi. Through this plant tissue feeding, the root cortex harboring the mycorrhizal fungi often gets destroyed and the establishment of fungus association is hampered (Talavera et al., 2001; Hol and Cook, 2005) (Table 1).

**Table 1:** Comprehensive overview on the effects of mycorrhizal fungi interactions with plants and development of diseases by phytopathogens.

**Arbuscular mycorrhizal fungi**	**Plants**	**Pathogens**	**Observations on disease incidence**	**References**
*Gigaspora albida, Paraglomus occultum, Acaulospora splendida, Funneliformis mosseae*	Maize	Nematodes	Arbuscular mycorrhizal fungal root colonization resulted in high root infection by nematodes.	Alvarado-Herrejón et al., 2019
*Glomus fasciculatus*	Tomato	*Meloidogyne incognita* and *M. javanica*	Mycorrhizal fungal colonization of tomato plants in the presence of root knot nematodes increased the incidence of the disease and gall development	Bagyaraj et al., 1979
*Glomus intraradices*	*Lycopersicon esculentum* (Tomato)	Tobacco mosaic virus	Root colonization by *G. intraradices* significantly increased viral disease incidence and severity in the tomato plants.	Столярчукet al., 2009
*Funneliformis coronatum, Claroideoglomus entunicatum, F. mosseae* and *Rhizophagus irregularis*	Wheat (*Triticum aestivum*)	*Pratylenchus neglectus* (nematode)	Arbuscular mycorrhizal fungal colonization of wheat plants significantly increased the nematodes population by 47 to 117 percent.	Frew et al., 2018
*Glomus intraradices*	Potato	Potato virus Y	Potato plants colonized by arbuscular mycorrhizal fungi have increased disease severity than the un-colonized ones	Sipahioglu et al., 2009
*Glomus intraradices*	Tobacco (*Nicotiana tabacum* cv. *Xanthinc*)	*Botrytis cinerea*, Tobacco mosaic virus	High disease severity and incidence were observed in tobacco plants colonized by the mycorrhizal fungi	Shaul et al., 1999
*Glomus sp*.	Tomato	Tobacco mosaic virus	Mycorrhizal fungi infection of tomato plants significantly increased the viral titers in the infected plants	Jabaji-Hare and Stobbs, 1984
*Funneliformis macrocarpa*	Tomato, Petunia, Strawberry	Tomato aucuba mosaic virus, Potato virus X, Arabis mosaic virus	Mycorrhizal fungi colonization of these plants enhanced increase in the viral titer of the infected plants and its associated diseases	Daft and Okusanya, 1973
*Glomus constrictum*	Bean (*Phaseolus vulgaris* L.)	*Uromyces appendiculatus*	It increased the incidence of rust disease and the fungal infection	Meyer and Dehne, 1986
*Rhizophagus clarus, Claroideoglomus etunicatum*	*Cymbopogon citratus* (lemongrass)	*Pratylenchus brachyurus*	An increase of 8.6 times in nematodes population was observed in the mycorrhizal fungi-colonized plants compared with the non-colonized ones	e Silva et al., 2021

Inoculation of plants with mycorrhizal fungi and pathogenic organisms proves to contribute to the development of disease severity in plants infected with pathogens without mycorrhizal fungi. Camprubi et al. (2020), in a greenhouse study, observed that loquat plants (*Eriobotrya japonica*) treated with arbuscular mycorrhizal fungi (*R. irregulare* and a native AMF isolate from loquat soils) and white root rot pathogenic fungi (*Armillaria mellea*) came down with visible necrotic lesions and severe disease symptoms more than the control (loquat plant and the pathogen). The mycorrhizal fungi were found to improve the plants' vegetative growth and properties, despite the presence of the pathogens and disease symptoms, giving the plants tolerance to the pathogens. This observation could be best described as mycorrhizal-enhanced compensation to pathogens induced damage. Through nutrient uptake and root aided functions, the loss of functionality of pathogen-infected roots is compensated by mycorrhizal fungi symbiotic effects. The modulation of plant defense hormones, reactive oxygen species production, fucose, UDP-glucose, ADP-glucose, and reduced accumulation of fatty acid, lipids, and phenolics were the various strategies employed by the mycorrhizal fungi to increase disease severity in the pathogen-infected plants (Harrier and Watson, 2004; Camprubi et al., 2020). Furthermore, it was observed by the authors that plants treated with native mycorrhizal fungi in the presence of the pathogenic fungi showed no disease damage in the plant shoot, whereas the plants treated with *R. irregulare* failed to prevent disease damage by the fungi. Differences in the mycorrhizal fungus effects were not accounted for. Several questions need to be resolved: do the native mycorrhizal fungi, in the course of evolution, acquire genes for the production of anti-fungal metabolites? What is the nutrient acquisition level of the native mycorrhizal fungi? Are jasmonic acid and ethylene defense chemicals, which are triggered in the presence of necrotrophic pathogen, weakly modulated by the native mycorrhizal fungi? Do the native mycorrhizal fungi grow faster than the white root rot pathogenic fungi (*Armillaria mellea*)? What are the contributions of bacterial symbionts of mycorrhizal fungi in fungal physiology and ecosystem services to the plants? These unanswered questions should form the subject of further discussions and research.

In another study, the incidence and severity of disease (necrotic lesion) were found to be higher in tobacco plants colonized by *G. intraradices* in the presence of tobacco mosaic virus or *Botrytis cinerea* pathogens. The leaves of these plants have higher necrotic lesions than the pathogen-infected control plants without mycorrhizal fungi. Plants would naturally employ their innate defense mechanisms to combat pathogenic organisms through the activation of defense signaling pathways and the production of salicylic acid, jasmonic acid, ethylene, and other antimicrobial substances as well as callose formation. However, when mycorrhizal fungi are involved in the relationship, the establishment of the fungi will lead to the modulation and subverting of the activation of the pathogenesis-related genes in the host. This event will increase the susceptibility level of the plants to come down with disease severity and symptoms in the presence of phytopathogens (Shaul et al., 1999). The interconnectedness of the plant immune system makes it possible for developing foliar pathogens and their associated disease establishment becomes more severe when pathogenesis-related genes are suppressed in the host roots by mycorrhizal fungi. Mycorrhizal fungi, though, could trigger pattern-triggered immunity at the earlier stage of infection, but this immunity is suppressed during the establishment of fungi symbiosis with the plants as describe previously. Treatment of avocado, citrus, and alfalfa plants with *G. fasciculatus*, and phytophthora pathogens (*Phytophthora parasitica, P. cinnamomi, and P. megasperma*) increased the severity of the disease caused by the pathogens on the infected plants as reported by Davis et al. (1978). Also affected was the mycorrhizal fungus sporulation, which was retarded in the presence of the pathogens, showing that the pathogen must have interfered with the proper establishment of the mycorrhizal fungal symbiosis with the host plant either by destroying the fungi attached to the root cortex, out competing with the mycorrhizal fungi for nutrients and attachment surfaces, or secretion of antagonistic chemicals. Therefore, adequate understanding of complete physiological changes in plants during root colonization by the mycorrhizal fungi in field settings and under the influence of atmospheric conditions is required. This will explain if environmental conditions exert any influence in mycorrhizal symbiosis with plants under the influence of pathogen attack or if plant physiological responses are better in the greenhouse than in the field or vice versa. Studies should focus on understanding the ecology and evolution of mycorrhizal fungi and its bacterial symbiont interactions in relation to plant–pathogen interactions under mycorrhizal fungal symbiosis.

**Figure 6:**
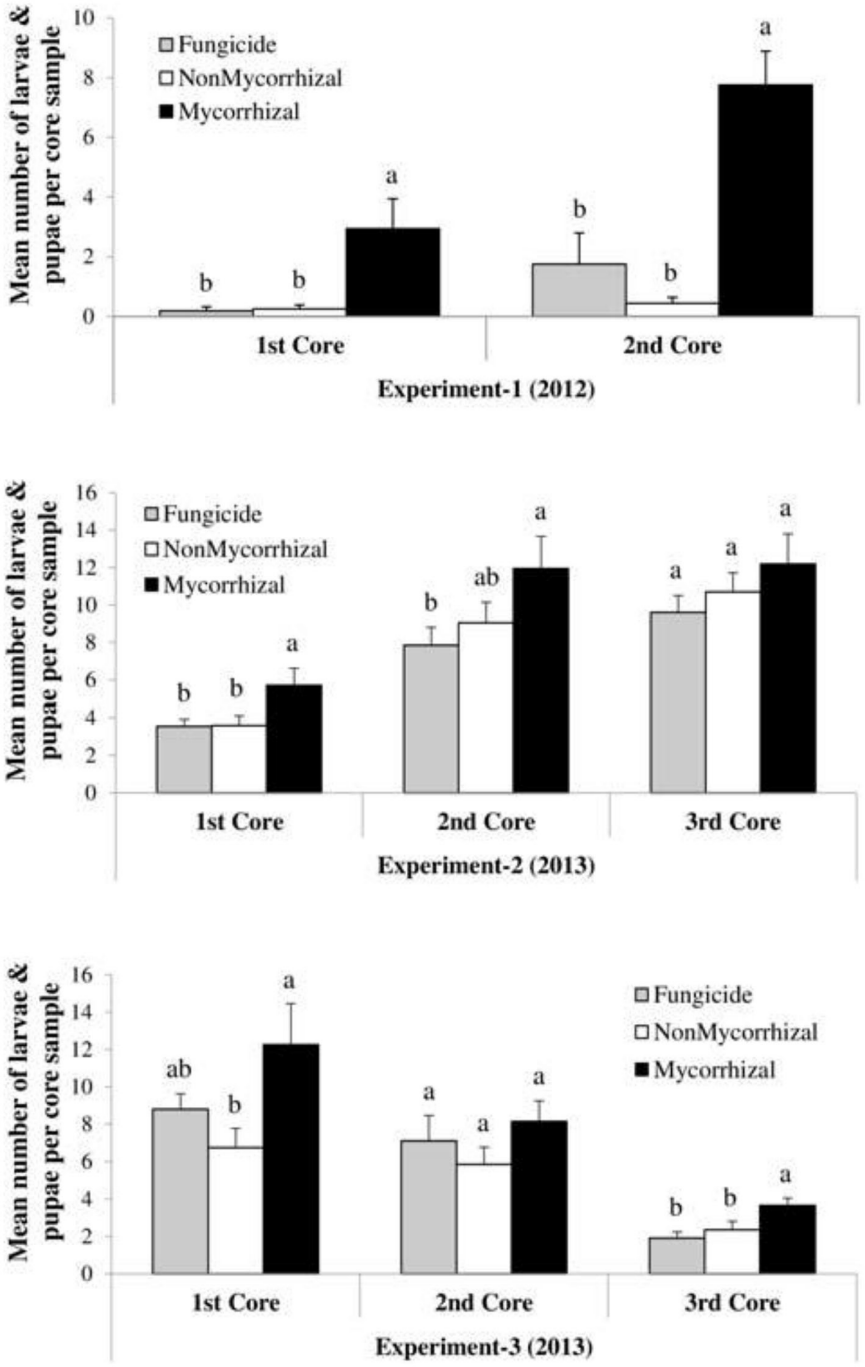
Case study on the effects of arbuscular mycorrhizal fungi treatments on the densities (larvae and pupae per core sample) of *Lissorhoptrus oryzophilus* (± SE) in three field experiments (Experiment-1, Experiment-2, and Experiment-3) during 2012 and 2013. Fungicide: rice seeds + fungicides + sterilized AMF, non-mycorrhizal: rice seeds + sterilized AMF, and mycorrhizal: rice seeds + live AMF. Bars and lower case letters at the column head indicate that means differ significantly (LSD, *P* ≤ 0.05). “The core samples are gotten by taking root/soil core samples from each plot using core sampler of metal cylindrical diameter of 9.2 cm and a depth of 7.6 cm.” Adapted from Bernaola et al. (2018) published by Frontiers in Plant Science in 2018 with CC BY license permission.

Nevertheless, microbial pathogens are not the only beneficially of mycorrhizal fungal suppression of plant immunity; pests, on the other hand, are equally strong beneficially too. The degree of mycorrhizal fungi colonization of plants has been correlated with the gain in body mass of leaf feeding insect pest. At low mycorrhizal colonization, the body mass of the pest is small, but as time increased and the rate of colonization increased too, the body mass of the insect increased significantly. This could be explained by the Bennett et al. (2006) hypothesis on nutrient quality and quantity influenced by mycorrhizal symbiosis with the associated plants. The more the quality and quantity of plant nutrients and biomass, the more the available nutrients and biomass to be consumed by leaf-chewing insect herbivory. This was reported by Schoenherr (2017) who noticed that potato plants, colonized by *G. intraradices*, were found to exert varying effects on cabbage looper *Lepidopteran* larvae feeding on potato plant leaves. While measuring the biomass of the insect larvae, it was observed that at low colonization of the plant roots by the fungi, the insect biomass gain (i.e., weight) was low compared to when the fungus colonization was high. High levels of mycorrhizal fungi colonization correspond to an equal increase in insect biomass and body weight. Increase in shoot biomass in a study by Bernaola et al. (2018) noted nutrient composition to be the rationale behind the increase in plant susceptibility to feeding by *Lissorhoptrus oryzophilus* kuschel (rice water weevil) and *Spodoptera frugiperda* (fall armyworm) (Figure 6). The number of insect larvae and pupae numbers were significantly high in mycorrhizal plants than the non-mycorrhizal ones.

Mycorrhizal fungi colonization of the rice plants increased the plant biomass, which have corresponding increased effects on the population of these pests and their overall performance. The increased susceptibility of the plants to pest infestation, due to mycorrhizal colonization, could equally be attributed to the suppression of plant immunity by the fungi, thus altering jasmonic and salicylic acid signaling pathways in the colonized plants (Jung et al., 2012; Roger et al., 2013). Though, other factors, in addition to the plant biomass quality increased by mycorrhization, could play a role in plant susceptibility to insect herbivory, factors such as plant variety/species, insect species involved, and environmental conditions (Koricheva et al., 2009; Pineda et al., 2010) as well as mycorrhizal fungi inoculum concentration (Eichholtzer et al., 2021) determine the outcome of the pest herbivory effects on plants.

A more critical determinant of pest herbivory is the jasmonic acid concentration in the plants. Recall again that successful colonization of plant roots and activation of arbuscular mycorrhization 2 (RAM2) required for mycorrhizal fungus penetration of plant roots are dependent on jasmonic acid signaling pathway. This signaling pathway is recruited by the fungi to establish a symbiotic relationship with the plants (Jacobs et al., 2011; Liu et al., 2022). The same applies to pest herbivory and performance. A study by Grover et al. (2022) has shown that sorghum plants defective in jasmonic acid signaling and its production reduced deterred aphids (*Melanaphis sacchari*) feeding from the plants phloem sap. This deterring effect on aphid feeding and reproduction was restored when the plants were treated with exogenous jasmonic acid. Jasmonic acid, therefore, enhances aphid proliferation, reproduction, and colonization of the plants.

Increase in survival of insect larvae, body weight, and overall performance, including its oviposition on mycorrhizal fungi-colonized plants, have been reported in several studies (Goverde et al., 2000; Gange et al., 2002; Hoffmann et al., 2009; Cosme et al., 2011; Currie et al., 2011). This could be associated with an increase in the phosphorus and nitrogen contents of the plant tissue. Thus, nutritional quantity and quality hypothesis applies to pest selection and invasion of plants colonized by mycorrhizal fungi. To support this idea, Zeng et al. (2022) reported that *Medicago truncatula* plants defective in H^+^-ATPase genes that is required for efficient transport and exchange of phosphorus through mycorrhizal nutrient uptake channel was weak in the enhancement of pest herbivory performance. Surprisingly, plants colonized by mycorrhizal fungi with intact phosphate transport genes (H^+^-ATPase) showed a significant increase in *Spodoptera exigua* herbivory performance. Additionally, plant inoculation with mycorrhizal fungi gave rise to increase in spider mite (*Tetranychus urticae*) feeding and reproduction on infested bean plants (*Phaseolus vulgaris*) (Khaitov et al., 2015). This is a function of plants that improved nutritional quality by the fungi, which enhances the plant sap contents that the pests feed on by piercing and sucking the sap from the plant parenchyma tissues (Patiño-Ruiz and Schausberger, 2014). These nutrients are required for the pests' reproduction and physiological activities. Therefore, mycorrhizal fungi colonization of plants influences the ground herbivorous insect pest community (Table 2). The observation from Table 2 according to Qu et al. (2023) showed that the effect of mycorrhizal fungi colonization of plants on the abundance of herbivory insects is dependent of soil fertility and water availability. However, the presence of water is the most critical factor that influences herbivory abundance on arbuscular mycorrhizal fungi-colonized plants.

**Table 2:** Outlook on the impact of insect herbivory effects on plants colonized by mycorrhizal fungi.

**Arbuscular mycorrhizal fungi**	**Plants**	**Insect Herbivore**	**Observations**	**References**
*Funneliformis mosseae*	*Artemisia ordosica*	*Chrysolina aeruginosa*	Mycorrhizal fungal colonization of plants enhanced the insect herbivore abundance and activities	Qu et al., 2023
*Glomus microaggregatum, Rhizophagus irregularis, Sclerocystis dussii, Glomus deserticola, Funneliformis mosseae, Rhizophagus fasciculatum*	Rice (*Oryza sativa* L.)	Rice water weevil (*Lissorhoptrus oryzophilus*), Fall armyworm (*Spodoptera frugiperda*)	Colonization of rice plants by arbuscular mycorrhizal fungi promoted plants susceptibility to the pests, increased their growth and population densities	Bernaola and Stout, 2019
Arbuscular mycorrhizal fungi	*Plantago lanceolata*	Glanville fritillary butterfly (*Melitaea cinxia* L.)	Mycorrhizal fungal colonization of the plants roots enhanced the insect herbivory activities	Rasmussen et al., 2017
*Claroideoglomus claroideum, Entrophospora infrequens, Racocetra fulgida, Funneliformis mosseae*	*Solanum lycopersicon* (tomato)	Beetle larvae (*Leptinotarsa decemlineata*)	Beetle herbivory activities were increased in the presence of mycorrhizal fungal colonization of the plants	Malik et al., 2018

## Conclusion

We posit that mycorrhizal fungal association with plants affects plant susceptibility to pathogenic microbes and herbivorous pests by altering the defense signaling traits of the colonized plants. This is perhaps obviously clear in the pathogenicity of biotrophic and necrotrophic pathogens and herbivorous pests of agricultural crops. Mycorrhizal fungi over the years have evolved specific means of subverting plant immune response or recruitment of jasmonic acid signaling pathway in establishing symbiotic relationship with their host plants. This fungi–plant immune subverting strategy underlies the remarkable consequences of increasing disease incidence in crops and pest infestation, which could result in dwindling plant yield and productivity and may, in the long term, lead to a decrease in food supply. The ways in which mycorrhizal fungi and plants are evolving to ensure balance while increasing the ecological services rendered to the plants by mycorrhizal fungi produce distinct regulatory channels that favor plant control of over-colonization by the mycorrhizal fungi. To ensure mycorrhizal plant control with pathogen infection, we recognized that pre-inoculation of the plants before exposing them to the pathogens could enhance the fungi biocontrol potential. However, this could not easily be applied in the field since pathogenic microbes are natural inhabitant of agricultural soil. Therefore, we recommend that mycorrhizal fungi be introduced alongside bacteria or fungi biocontrol organisms to enhance the tripartite plants-mycorrhizal fungi biocontrol microbes' symbiosis and control pest infestation.

## Author contributions

Both authors listed have made a substantial, direct, and intellectual contribution to the work and approved it for publication.
