# Supplementary material for: Susceptibility and plant immune control—a case of mycorrhizal strategy for plant colonization, symbiosis, and plant immune suppression
Source: Front Microbiol. 2023 Jul 5;14:1178258. doi: 10.3389/fmicb.2023.1178258 (PMC10355322; doi:10.3389/fmicb.2023.1178258)
Supplement: Supplementary file 1 [file Data_Sheet_1.docx]

Susceptibility and plant immune control – a case of mycorrhizal strategy for plant colonization, symbiosis and plant immune suppression.

Matthew Chekwube Enebe^1*^, Mariana Erasmus^1^

^1^Centre for Mineral Biogeochemistry, University of the Free State, Bloemfontein 9031, South Africa, [erasm@ufs.ac.za](mailto:erasm@ufs.ac.za), [enebematthew@gmail.com](mailto:enebematthew@gmail.com)

*Correspondence: [enebematthew@gmail.com](mailto:enebematthew@gmail.com)

Supplementary Table

Table S1. Trend in research on the role of arbuscular mycorrhizal fungal colonization on plants susceptibility to phytopathogens and herbivores infestation. This table was used in plotting figure 3 contained in the main text.

| Publication year | Viral pathogens | Bacterial pathogens | Fungal pathogens | Oomycetes | Nematodes | Herbivores | References |
| --- | --- | --- | --- | --- | --- | --- | --- |
| 2009 | 2 | - | - | - | - | - | (Sipahioglu et al., 2009; Столярчук et al., 2009) |
| 2019 | - | - | - | - | 1 | 1 | (Alvarado-Herrejón et al., 2019; Bernaola and Stout, 2019) |
| 2018 | - | - | 1 | - | - | 3 | (Bernaola et al., 2018; Frew et al., 2018; Malik et al., 2018) |
| 2017 | - | - | - | - | - | 1 | (Rasmussen et al., 2017) |
| 2023 | - | - | - | - | - | 1 | (QU et al., 2023) |
| 2016 | - | 1 | - | - | - | - | (Malik et al., 2016) |
| 2011 | 1 | - | - | - | - | - | (Miozzi et al., 2011) |
| 1999 | 1 | - | 1 | - | - | - | (Shaul et al., 1999) |
| 1973 | 1 | - | - | - | - | - | (Daft and Okusanya, 1973) |
| 1984 | 1 | - | - | - | - | - | (Jabaji-Hare and Stobbs, 1984) |
| 2021 | - | - | - | - | - | 2 | (e Silva et al., 2021; Eichholtzer et al., 2021) |
| 1986 | - | - | 1 | - | - | - | (Meyer and Dehne, 1986) |
| 2012 | - | - | 1 | - | - | - | (Patale and Shinde, 2012) |
| 2013 | 1 | - | - | - | - | - | (Rúa et al., 2013) |
| 1978 | - | - | - | 1 | - | - | (Davis et al., 1978) |
| 2015 | - | - | - | - | - | 1 | (Khaitov et al., 2015) |
| 2020 | - | - | 1 | - | - | - | (Camprubi et al., 2020) |

References

Alvarado-Herrejón, M., Larsen, J., Gavito, M.E., Jaramillo-López, P.F., Vestberg, M., Martínez-Trujillo, M., Carreón-Abud, Y., 2019. Relation between arbuscular mycorrhizal fungi, root-lesion nematodes and soil characteristics in maize agroecosystems. Applied soil ecology 135, 1-8.

Bernaola, L., Cosme, M., Schneider, R.W., Stout, M., 2018. Belowground inoculation with arbuscular mycorrhizal fungi increases local and systemic susceptibility of rice plants to different pest organisms. Frontiers in Plant Science 9, 747.

Bernaola, L., Stout, M.J., 2019. Effects of arbuscular mycorrhizal fungi on rice-herbivore interactions are soil-dependent. Scientific reports 9, 1-12.

Camprubi, A., Solari, J., Bonini, P., Garcia-Figueres, F., Colosimo, F., Cirino, V., Lucini, L., Calvet, C., 2020. Plant performance and metabolomic profile of loquat in response to mycorrhizal inoculation, armillaria mellea and their interaction. Agronomy 10, 899.

Daft, M., Okusanya, B., 1973. Effect of endogone mycorrhiza on plant growth v. influence of infection on the multiplication of viruses in tomato, petunia and strawberry. New Phytologist 72, 975-983.

Davis, R., Menge, J., Zentmyer, G., 1978. Influence of vesicular-arbuscular mycorrhizae on Phytophthora root rot of three crop plants. Phytopathology 68, 1614-1617.

e Silva, M.T.R., Calandrelli, A., Rinaldi, L.K., Miamoto, A., Moreno, B.P., da Costa, W.F., Silva, C., Alberton, O., Dias-Arieira, C.R., 2021. Arbuscular mycorrhizae maintain lemongrass citral levels and mitigate resistance despite root lesion nematode infection. Rhizosphere 19, 100359.

Eichholtzer, J., Ballina-Gómez, H.S., Gómez-Tec, K., Medina-Dzul, K., 2021. Arbuscular mycorrhizal fungi influence whitefly abundance by modifying habanero pepper tolerance to herbivory. Arthropod-Plant Interactions 15, 861-874.

Frew, A., Powell, J.R., Glauser, G., Bennett, A.E., Johnson, S.N., 2018. Mycorrhizal fungi enhance nutrient uptake but disarm defences in plant roots, promoting plant-parasitic nematode populations. Soil Biology and Biochemistry 126, 123-132.

Jabaji-Hare, S., Stobbs, L., 1984. Electron microscopic examination of tomato roots coinfected with Glomus sp. and tobacco mosaic virus. Phytopathology 74, 277-279.

Khaitov, B., Patiño‐Ruiz, J.D., Pina, T., Schausberger, P., 2015. Interrelated effects of mycorrhiza and free‐living nitrogen fixers cascade up to aboveground herbivores. Ecology and evolution 5, 3756-3768.

Malik, R.J., Ali, J.G., Bever, J.D., 2018. Mycorrhizal composition influences plant anatomical defense and impacts herbivore growth and survival in a life-stage dependent manner. Pedobiologia 66, 29-35.

Malik, R.J., Dixon, M.H., Bever, J.D., 2016. Mycorrhizal composition can predict foliar pathogen colonization in soybean. Biological control 103, 46-53.

Meyer, J., Dehne, H., 1986. The influence of VA mycorrhizae on biotrophic leaf pathogens [Glomus etunicatus, Glomus constrictus; Bremia lactucae, Uromyces phaseoli], Physiological and Genetical Aspects of Mycorrhizae. Aspects physiologiques et genetiques des mycorhizes, Dijon (France), 1-5 Jul 1985. INRA.

Miozzi, L., Catoni, M., Fiorilli, V., Mullineaux, P.M., Accotto, G.P., Lanfranco, L., 2011. Arbuscular mycorrhizal symbiosis limits foliar transcriptional responses to viral infection and favors long-term virus accumulation. Molecular plant-microbe interactions 24, 1562-1572.

Patale, S., Shinde, B., 2012. Influence of Glomus species and soil phophorous on Verticillium wilt in Bt Cotton. J. Adv. Lab. Res 3, 307-312.

QU, L., Wang, M., Biere, A., 2023. Effects of arbuscular mycorrhizal fungi on plant growth and herbivore infestation depend on availability of soil water and nutrients. Frontiers in Plant Science 14, 167.

Rasmussen, P.U., Amin, T., Bennett, A.E., Karlsson Green, K., Timonen, S., Van Nouhuys, S., Tack, A.J., 2017. Plant and insect genetic variation mediate the impact of arbuscular mycorrhizal fungi on a natural plant–herbivore interaction. Ecological Entomology 42, 793-802.

Rúa, M.A., Umbanhowar, J., Hu, S., Burkey, K.O., Mitchell, C.E., 2013. Elevated CO 2 spurs reciprocal positive effects between a plant virus and an arbuscular mycorrhizal fungus. New Phytologist 199, 541-549.

Shaul, O., Galili, S., Volpin, H., Ginzberg, I., Elad, Y., Chet, I., Kapulnik, Y., 1999. Mycorrhiza-induced changes in disease severity and PR protein expression in tobacco leaves. Molecular Plant-Microbe Interactions 12, 1000-1007.

Sipahioglu, M.H., Demir, S., Usta, M., Akkopru, A., 2009. Biological relationship of Potato virus Y and arbuscular mycorrhizal fungus Glomus intraradices in potato. Pest Tech 3, 63-66.

Столярчук, I., Шевченко, Т., Поліщук, В., Кріпка, А., 2009. Virus infection course in different plant species under influenceof arbuscular mycorrhiza. Мікробіологія і біотехнологія, 70-75.
